# Multiscale Models to Evaluate the Impact of Chemical Compositions and Test Conditions on the Mechanical Properties of Cement Mortar for Tile Adhesive Applications

**DOI:** 10.3390/ma17153807

**Published:** 2024-08-01

**Authors:** Warzer Mohammed-Sarwar Qadir, Serwan Khurshid Rafiq Al Zahawi, Ahmed Salih Mohammed

**Affiliations:** Civil Engineering Department, College of Engineering, University of Sulaimani, Kurdistan 46001, Iraq; warzer.sarwar@univsul.edu.iq (W.M.-S.Q.); serwan.rafiq@univsul.edu.iq (S.K.R.A.Z.)

**Keywords:** water/cement, cement/sand ratio, pH curing, chemical composition, mechanical properties, modeling

## Abstract

This study aims to develop systematic multiscale models to accurately predict the compressive strength of cement mortar for tile adhesive applications, specifically tailored for applications in the construction industry. Drawing on data from 200 cement mortar tests conducted in previous studies, various factors such as cement/water ratios, curing times, cement/sand ratios, and chemical compositions were analyzed through static modeling techniques. The model selection involved utilizing various approaches, including linear regression, pure quadratic, interaction, M5P tree, and artificial neural network models to identify the most influential parameters affecting mortar strength. The analysis considered the water/cement ratio, testing ages, cement/sand ratio, and chemical compositions, such as silicon dioxide, calcium dioxide, iron (III) oxide, aluminum oxide, and the pH value. Evaluation metrics, such as the determination coefficient, mean absolute error, root-mean-square error, objective function, scatter index, and a-20 index, were employed to ensure the accuracy of the compressive strength estimates. Additionally, empirical equations were utilized to predict flexural and tensile strengths based on the compressive strength of the cement mortar for tile adhesive applications.

## 1. Introduction

Cement mortar comprises cementitious substances, fine materials, and water, which can exist in either a soft or solid state [1]. Mortar is an adhesive that fills the space between construction elements and is used for decorative purposes. Cement mortar possesses three essential characteristics: workability, strength, and durability [2]. Haach et al. conducted a study in 2011 to explore how the variation in sand grading and the water/cement (w/c) ratio impacts both the fluidity and compressive strength (CS) of cement mortar [3]. It was found that as the w/c ratio increases, the mechanical characteristics of the mortar decrease, but the ease of handling rises [3]. Materials based on ordinary Portland cement (OPC) have been extensively utilized and remain a popular choice for construction since its inception in the 18th century; currently, concrete and cement mortar are perceived as unsustainable and have limited compatibility with natural materials. Therefore, metamaterials like lime cement are being explored as alternatives [4]. The consistency of a mortar mixture in its raw or fresh state is crucial. The quantity of beneficial internal effort required to achieve complete compaction is a key aspect of this consistency. A mortar mixture that is easy to place and compress without segregation is considered manageable. This trait is essential as it is closely linked to both compaction and strength. The ease of handling varies across different varieties of mortar. Unlike a large concrete mass or cement mortar structure, higher manageability is necessary for thin, hard-to-reach, or substantially reinforced areas.

The compressive strength of hardened cement mortar is a crucial indicator of its quality and performance in construction projects. In addition, the properties of tension, flexural, and bond strengths show a corresponding improvement as the compressive strength increases [5]. The tensile strength reduces by 55–60% as the fineness modulus of sand transitions from 3.21 to 1.72 [6]. Mineral additives enhance hardened cement mortar performance, mechanical characteristics, and durability. In addition, the inclusion of mineral additives has the effect of decreasing the emission of carbon dioxide (CO_2_) and potentially mitigating the adverse environmental impact associated with cement production [7]. Various research studies have examined the elements that affect the tensile strength of cement mortar. The addition of micro- and nano-silica into cement mortar results in an improvement in its split tensile strength [8]. Speciality cement is being developed by grinding clinker with elastomer polymeric latexes, such as styrene-butadiene rubber (SBR) or polyvinyl acetate (PVA), to improve its tensile and bond properties. This specific kind of pre-mixed cement is necessary for adhesive applications needing more robust adherence to substrates. Some examples of these uses are tile adhesives and repairing mortars. Waterproofing slurries are necessary for improving the longevity and adhesion of repair and injection works on implanted steel bars. The test results demonstrated that latexes retain their efficacy even after undergoing grinding, leading to alterations in cement properties such as improved workability and better flexural and pull-off bond strengths. To avoid any detrimental effects on the ability of clinker to be ground, the inclusion rates of SBR and PVA should be limited to less than 0.4% and 0.3% of the total cement mass, respectively. The topic of discussion revolves around the use of polymer-modified cement for the manufacturing of tile adhesives according to the specifications outlined in the EN 12004 standard [9].

Various research studies investigated the mechanical properties of cement mortar [10,11,12]. The laboratory determines the cement mortar samples’ compressive, flexural, and tensile strengths by crushing standardized cylinders or cubes [13]. The experimental methodology complies with international standards. However, laboratory testing is considered inefficient and requires many resources, mostly because of its expensive and time-consuming nature. Currently, by harnessing the progress in artificial intelligence (AI), machine learning algorithms (ML) have recently become a new tool for forecasting particular mechanical characteristics of cement mortar [14,15]. Regression, clustering, and classification are machine learning techniques that may estimate many elements with varying levels of success. These techniques assist with effectively forecasting the compressive strength of cement mortar. Soft computing (SC) has been extensively researched and applied, particularly in structural engineering, due to its ability to tackle complicated issues, including uncertainty and different parameters. The characteristics of cement mortar can be modeled using several methods, such as computational modeling and statistical techniques, and developing tools like regression analysis, artificial neural networks (ANN), and the M5P-tree model [16].

The link between curing temperature and the compressive strength of cement mortar is intricate and influenced by multiple factors. Raising the temperature during the curing process can decrease the long-term compressive strength of mortar mixtures [17]. However, this impact is less significant in combinations that include fly ash. The elevated curing temperature can hurt the mechanical strength of specific cement asphalt mortars while enhancing the compressive strength of others [18].

The chemical composition of cement has a substantial influence on the strength of cement, mortar, and concrete. Exceeding a particular threshold of CaO content hinders its ability to react with other compounds due to free lime in the clinker [19]. The pH value of water used during the curing process can substantially affect the compressive strength of cement mortar and concrete. According to Wicaksono and Nurwidayati [20], acid water with low pH can reduce the compressive strength of concrete, particularly when exposed to wet–dry curing situations. Similarly, Dauda et al. noted a decrease in the compressive strength of concrete when it was cured in polluted water [21]. Moreover, a higher proportion of wastewater resulted in a more significant reduction in strength. However, according to Bediako et al., curing in lime-saturated water results in more significant strength growth in mortar specimens than in freshwater [22].

This study aims to evaluate the influence of the chemical composition of cement, pH levels, curing temperature, and mixture ratios on the compressive strength of cement mortar. Several modeling techniques, including linear regression, pure quadratic, interaction, M5P-tree, and ANN models, were utilized on a dataset of 200 samples collected from various research sources. The primary objectives are: (i) to perform statistical analysis to determine the impact of various mixture compositions, cement chemical composition, pH, and curing temperature on the compressive strength of cement mortar, (ii) to develop a reliable model for predicting the compressive strength of cement mortar using different accuracy performance metrics for the models’ prediction, and (iii) to identify the most accurate model for evaluating the compressive strength of cement mortar. The data gathered from previous research investigations is condensed and presented in Table 1. 

### The Novelty of the Study

1. The work presents a systematic multiscale modeling strategy to precisely forecast the compressive strength of cement mortar, particularly designed for tile adhesive applications in the construction sector;
2. This study employs data from 200 cement mortar experiments completed in prior research and thoroughly examines several parameters, such as cement/water ratios, curing durations, cement/sand ratios, and chemical compositions;
3. The study utilizes various modeling tools, including linear regression, pure quadratic models, interaction models, M5P trees, and artificial neural networks, to determine the most relevant elements that impact mortar strength. The variety of modeling approaches available contributes to a strong and reliable foundation for making predictions;
4. The examination includes comprehensive chemical compositions, such as silicon dioxide, calcium dioxide, iron (III) oxide, aluminum oxide, and pH value, contributing to a more profound comprehension of their influence on mortar strength;
5. Employing several assessment metrics, such as determination coefficient, mean absolute error, root-mean-square error, objective function, scatter index, and a-20 index, guarantees the precision and dependability of the compressive strength estimations;
6. The study forecasts the compressive strength and utilizes empirical equations to forecast the flexural and tensile strengths, which depend on cement mortar’s compressive strength. This comprehensive strategy greatly enhances the value of the building sector;
7. The research focuses on applying tile adhesive, a specialized and crucial aspect of the building industry. It addresses the unique requirements and difficulties connected with this field.

## 2. Material and Methods

**Figure 1:**
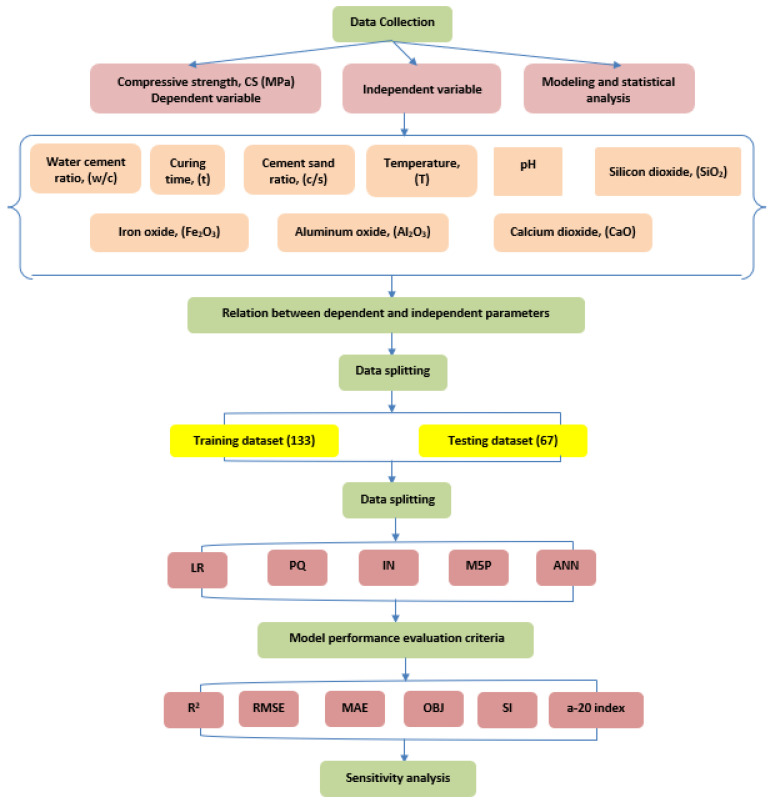
The flowchart diagram for the study.

The study examined the effects of various water-to-cement (w/c) ratios and curing durations of up to 365 days, as well as varied chemical compositions of cement and pH levels of curing water, on the compressive strength of cement mortar [7,23,24,25,26,27,28,29,32,33,34,35,36,37,38,39,40,41,42,43,44,45,46,47,48,49]. Silicon dioxide and calcium oxide levels were investigated, with silicon dioxide being tested at levels up to 30% and calcium oxide at levels up to 76.5%. The training dataset comprised 66% of the entire dataset and was utilized for developing the model, while the remaining subset was utilized for evaluating the model. The main input variables consisted of the water-to-cement ratio (w/c), curing time (t), cement-to-sand ratio (c/s), curing temperature (T), pH of the curing water (pH), and the chemical composition of the cement, with a particular focus on silicon dioxide (SiO_2_), calcium oxide (CaO), aluminum oxide (Al_2_O_3_), and iron (III) oxide (Fe_2_O_3_). During the modeling procedure, many statistical evaluations were used to evaluate the correctness of the model and determine the most dependable one. These assessments included the determination coefficient (R^2^), mean absolute error (MAE), root-mean-square Error (RMSE), objective function (OBJ), scatter index (SI), and a-20 index. In addition, the study employed two empirical equations to determine the flexural and tensile strengths by relying on the compressive strength. Figure 1 illustrates the study flow chart.

### 2.1. Modelling and Statistical Assessment

In this study, five models, which are linear regression, pure quadratic, interaction, M5P, and ANN models, were used to estimate the compressive strength of cement mortar as follows [50,51]:

(1) $$\begin{array}{cc} CS=\beta_{1}\left(w/b\right) & +\beta_{2}\left(t\right)+\beta_{3}\left(c/s\right)+\beta_{4}\left(T\right)+\beta_{5}\left(pH\right)+\beta_{6}\;\left(SiO_{2}\right)+\beta_{7}\left(CaO\right)\\ & +\beta_{8}\left({Al}_{2}O_{3}\right)+\beta_{9}\;({Fe}_{2}O_{3})+\beta_{10} \end{array}$$

(2) $$\begin{array}{cc} CS\;=\beta_{0}+\beta_{1}\left(w/b\right) & +\;\beta_{2}\left(t\right)+\beta_{3}\left(c/s\right)+\beta_{4}\left(T\right)+\beta_{5}\left(pH\right)+\beta_{6}\left(SiO_{2}\right)\\ & +\beta_{7}\left(CaO\right)+\beta_{8}\left({Al}_{2}O_{3}\right)+\beta_{9}\;({Fe}_{2}O_{3})+\beta_{11}(w/c)+\beta_{12}{\left(t\right)}^{2}\\ & +\beta_{13}\left(c/s\right)+\beta_{14}({T)}^{2}{+\;\beta_{15}\left(pH\right)}^{2}{+\;\beta_{16}\left(SiO_{2}\right)}^{2}+\beta_{17}(CaO)\\ & +\beta_{18}({{Al}_{2}O_{3})}^{2}+\beta_{19}({{Fe}_{2}O_{3})}^{2} \end{array}$$

(3) $$\begin{array}{cc} CS={\;\beta }_{1} & +\beta_{2}\left(w/c\right)+\beta_{3}\left(t\right)+\beta_{4}\left(c/s\right)+\beta_{5}\left(T\right)+{\;\beta }_{6}\left(pH\right)+{\;\beta }_{7}\left(SiO_{2}\right)+\beta_{8}\left(CaO\right)+{\;\beta }_{9}\left({Al}_{2}O_{3}\right)\\ & +{\;\beta }_{10}\left({Fe}_{2}O_{3}\right)+{\;\beta }_{11}\left(w/c\right)\left(t\right)+{\;\beta }_{12}\left(w/c\right)\left(c/s\right)+{\;\beta }_{13}\left(w/c\right)\left(T\right)+{\;\beta }_{14}\left(w/c\right)\left(pH\right)\\ & +{\;\beta }_{15}\left(w/c\right)\left(SiO_{2}\right)+{\;\beta }_{16}\left(w/c\right)\left(CaO\right)+{\;\beta }_{17}\left(w/c\right)\left({Al}_{2}O_{3}\right)+{\;\beta }_{18}\left(w/c\right)\left({Fe}_{2}O_{3}\right)+{\;\beta }_{19}\left(t\right)\left(c/s\right)\\ & +{\;\beta }_{20}\left(t\right)\left(T\right)+{\;\beta }_{21}\left(t\right)\left(pH\right)+{\;\beta }_{22}\left(t\right)\left(SiO_{2}\right)+{\;\beta }_{23}\left(t\right)\left(CaO\right)+{\;\beta }_{24}\left(t\right)\left({Al}_{2}O_{3}\right)\\ & +{\;\beta }_{25}\left(t\right)\left({Fe}_{2}O_{3}\right)+{\;\beta }_{26}\left(c/s\right)\left(T\right)+{\;\beta }_{27}\left(c/s\right)\left(pH\right)+\beta_{28}\left(c/s\right)\left(SiO_{2}\right)+{\;\beta }_{29}\left(c/s\right)\left(CaO\right)\\ & +{\;\beta }_{30}\left(c/s\right)\left({Al}_{2}O_{3}\right)+{\;\beta }_{31}\left(c/s\right)\left({Fe}_{2}O_{3}\right)+{\;\beta }_{32}\left(T\right)\left(pH\right)+{\;\beta }_{33}\left(T\right)\left(SiO_{2}\right)+{\;\beta }_{34}\left(T\right)\left(CaO\right)\\ & +{\;\beta }_{35}\left(T\right)\left({Al}_{2}O_{3}\right)+{\;\beta }_{36}\left(T\right)\left({Fe}_{2}O_{3}\right)+\beta_{37}\left(pH\right)\left(SiO_{2}\right)+{\;\beta }_{38}\left(pH\right)\left(CaO\right)+{\;\beta }_{39}\left(pH\right)\left({Al}_{2}O_{3}\right)\\ & +{\;\beta }_{40}\left(pH\right)\left({Fe}_{2}O_{3}\right)+{\;\beta }_{41}\left(SiO_{2}\right)\left(CaO\right)+{\;\beta }_{42}\left(SiO_{2}\right)\left({Al}_{2}O_{3}\right)+{\;\beta }_{43}\left(SiO_{2}\right)\left({Fe}_{2}O_{3}\right)\\ & +{\;\beta }_{44}\left(CaO\right)\left({Al}_{2}O_{3}\right)+{\;\beta }_{45}\left(CaO\right)\left({Fe}_{2}O_{3}\right)+\beta_{46}\left({Al}_{2}O_{3}\right)\left({Fe}_{2}O_{3}\right) \end{array}$$

The M5P-tree model, based on Quinlan’s M5 approach, is specifically designed to efficiently handle large datasets with many characteristics [1,30,31,50,51,52,53,54,55]. Each node in this model offers error estimates and provides specific information about the criteria employed for tree division. The evaluation of a node’s related function is determined by minimizing the expected error using the feature that results in the most significant decrease. The division criteria in the M5P tree are based on error estimations at the individual node level, where the node error is measured by its standard deviation. The splitting of nodes occurs depending on the property that achieves the lowest expected decrease in error. This branching leads to offspring nodes with a lower standard deviation. Parent nodes with larger sizes assess the available structures and choose the one most likely to reduce errors, resulting in a hierarchical structure that may promote overfitting.

Artificial neural networks (ANNs), opposite feed-forward neural networks, function based on different principles [56,57,58,59]. This system receives signals for processing in its input layer, one of its three main layers—input, hidden, and output. The output layer performs crucial functions such as making predictions and doing classifications. The essence of genuine computational artificial neural networks resides in their numerous intermediate layers positioned between the input and output. Data is transmitted from the input layer through the hidden layers to the output layer, replicating the data flow in a feed-forward network of artificial neural networks (ANNs). The model’s performance was enhanced through multiple trial cycles by optimizing the number of hidden layers. This optimization aimed at reducing errors and improving the R^2^ score. Equations (5) and (6) illustrate an artificial neural network (ANN) equation that consists of a single hidden layer [55,56,57,58,59].

(4) $$\beta_{n}=a_{n}\left(w/c\right)+b_{n}\left(t\right)+c_{n}\left(c/s\right)+d_{n}\left(T\right)+e_{n}\left(pH\right)+f_{n}\left(SiO_{2}\right)+g_{n}\left(CaO\right)+h_{n}\left({Fe}_{2}O_{3}\right)+i_{n}\left({Al}_{2}O_{3}\right)+i_{n}$$

(5) $$CS=\frac{{Node}_{1}}{1+e^{-\alpha 1}}+\frac{{Node}_{2}}{1+e^{-\alpha 2}}+\ldots +\frac{{Node}_{n}}{1+e^{-\alpha n}}+Treshold$$

Different statistical assessments were used to evaluate the performance of the developed models, such as CS, t, c/s, curing temperature, water pH, and the cement’s chemical composition [56,57,58,59]:

(6) $$RMSE=\sqrt{\frac{\sum_{i=1}^{n}{\left(yi-xi\right)}^{2}}{n}}$$

(7) $$R^{2}={\left(\frac{\;\sum_{i}(xi-\bar{x})*(yi-\bar{y})}{\sqrt{\sum_{i}{\left(xi-\bar{x}\right)}^{2}}*\sqrt{\sum_{i}{\left(yi-\bar{y}\right)}^{2}}}\right)}^{2}$$

(8) $$MAE=\frac{\sum_{i=1}^{n}{\left(yi-xi\right)}^{2}}{n}$$

(9) $$OBJ=\left(\frac{n_{tr}}{n_{all}}*\frac{{{RMSE}_{tr}+\;MAE}_{tr}}{R_{tr}^{2}+1}\right)+\left(\frac{n_{tst}}{n_{all}}*\frac{{{RMSE}_{tst}+\;MAE}_{tst}}{R_{tst}^{2}+1}\right)$$

(10) $$SI=\frac{RMSE}{yi}$$

(11) $$a20\;index\;(\%)=\frac{\sum Ymeasured/Ypredicted}{N}$$

### 2.2. Statistical Evaluation

**Figure 2:**
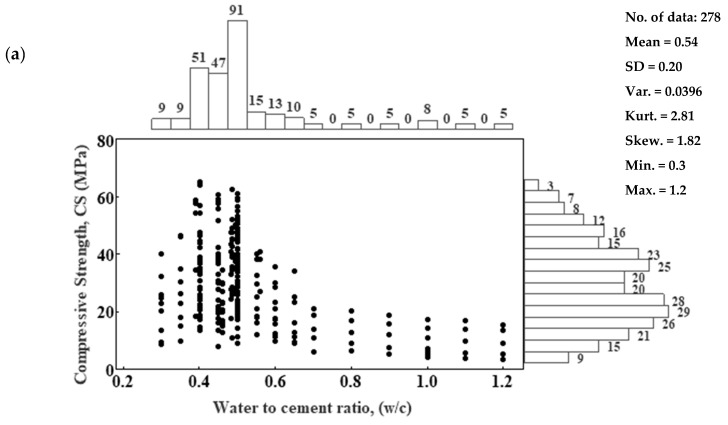

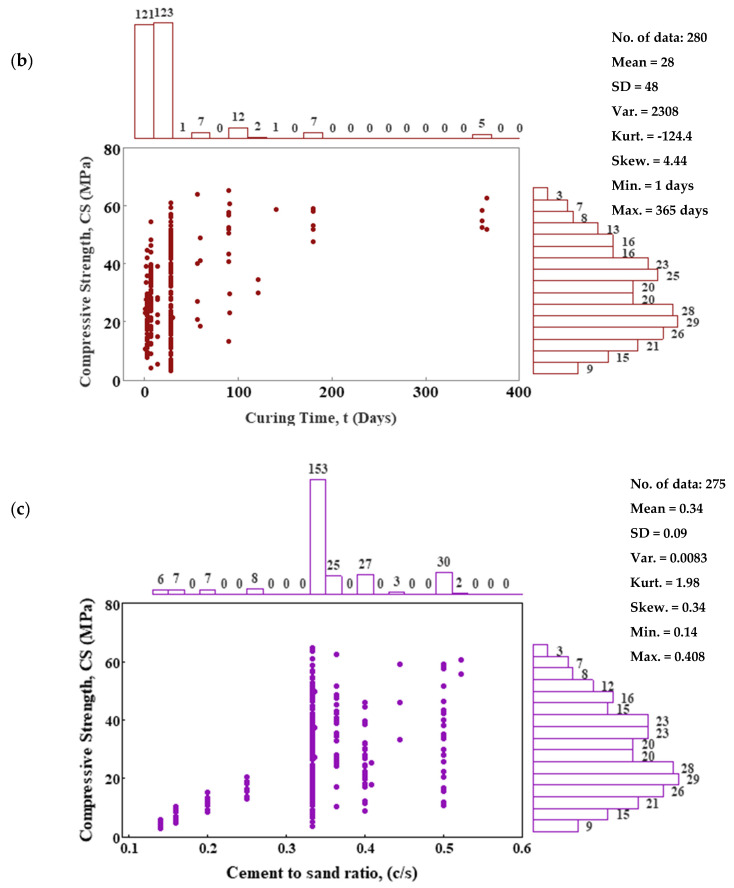

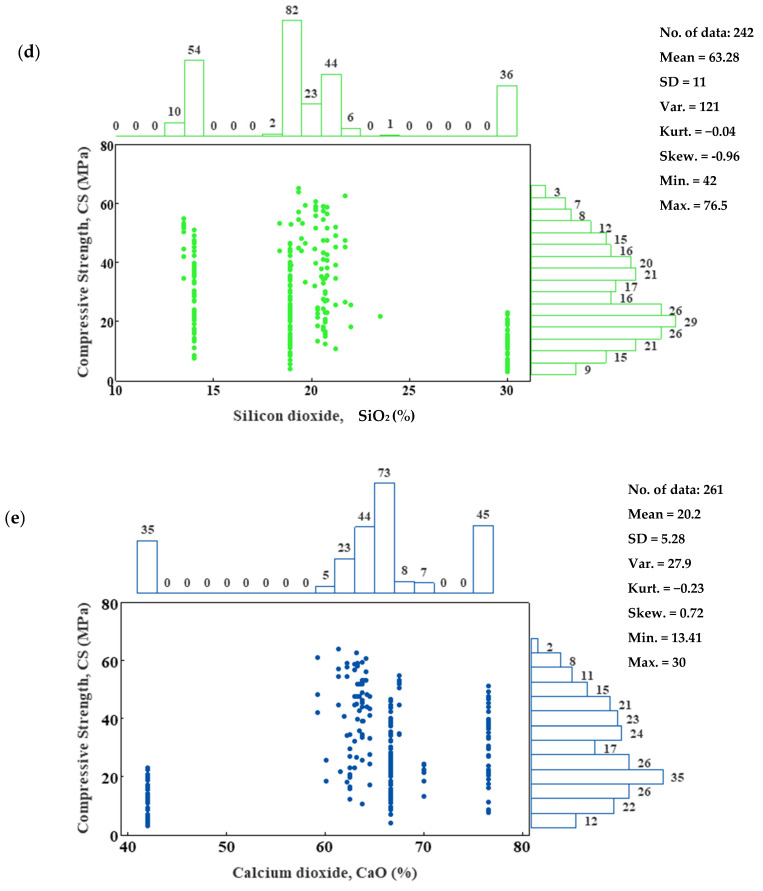

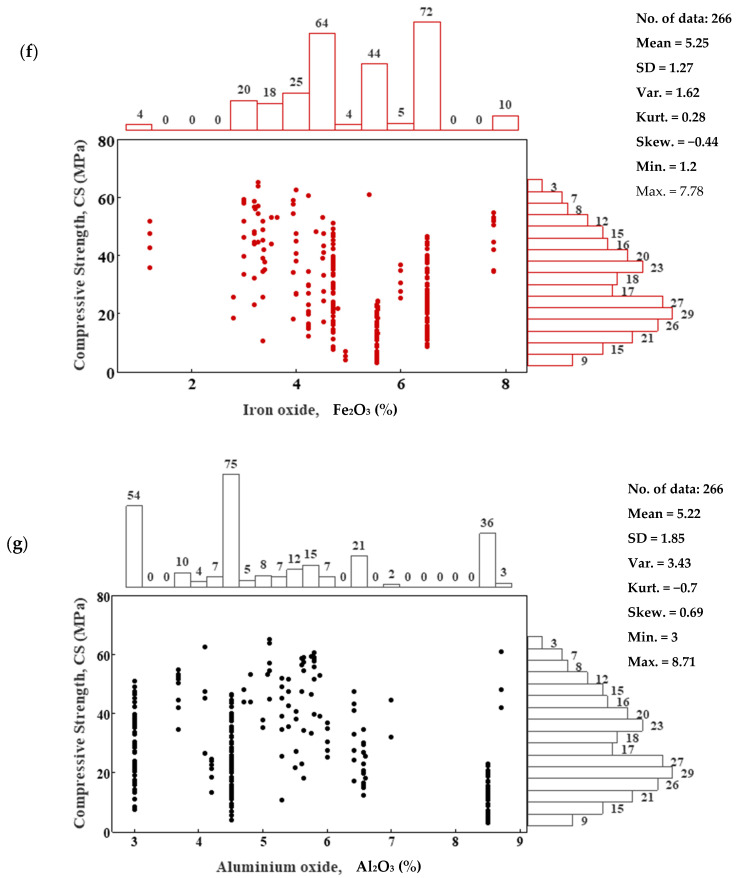
The marginal plot illustrates the relationship between the compressive strength of cement mortar and (**a**) w/c, (**b**) t (days), (**c**) c/s, (**d**) SiO_2_ (%), (**e**) CaO (%), (**f**) Fe_2_O_3_ (%), and (**g**) Al_2_O_3_ (%).

A statistical analysis was performed to identify the input parameters that impact the compressive strength of cement mortar. The marginal plot of the input variable of the cement mortar with the compressive strength is shown in Figure 2. Thus, the plots of all considered parameters of the compressive strength of cement mortar, including (a) w/c ratios, (b) curing time, (c) cement/sand ratio, (d) SiO_2_, (e) CaO, (f) Fe_2_O_3_, and (g) Al_2_O_3_, are shown in Figure 2.

The w/c ratio of cement mortar mixtures ranged from 0.30 to 1.2, and they were tested from an early age up to 365 days with a c/s ratio from 0.14 to 0.408.

The chemical composition of the cement also varied across the studies, with SiO_2_ content ranging from 13.48% to 20%, CaO content ranging from 42% to 76.5%, Fe_2_O_3_ content ranging from 1.2% to 7.78%, and Al_2_O_3_ content ranging from 3% to 8.71%. Table 2 summarizes the statistical analysis.

## 3. Measured and Predicted Compressive Strength

**Figure 3:**
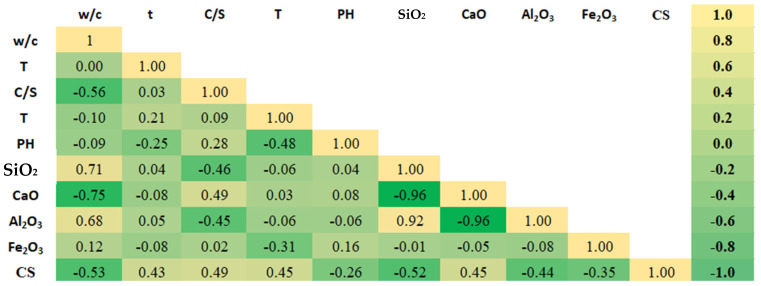
The correlation matrix presents the correlation coefficients between the dependent and independent variables.

As shown in Figure 3, no direct correlations were observed between the input variables and the compressive strength of cement mortar (output variable).

### 3.1. Linear Model

To model the connection between the CS of cement mortar and w/c, t, curing temperature, pH, and the cement chemical composition and to find the impact of the dependent variables on the cement mortar compressive strength, the linear model (Equation (12)) was developed.

(12) $$\begin{array}{cc} CS=-21.5\left(w/c\right) & +\;0.084\left(t\right)+45.4\left(c/s\right)+0.85\left(T\right)-0.56\;\left(pH\right)-2.9\;\left({SiO}_{2}\right)-2.19\;\left(CaO\right)-5.68\;\left({Al}_{2}O_{3}\right)\\ & -4.75\;\left({Fe}_{2}O_{3}\right)-259.8 \end{array}$$

**Figure 4:**
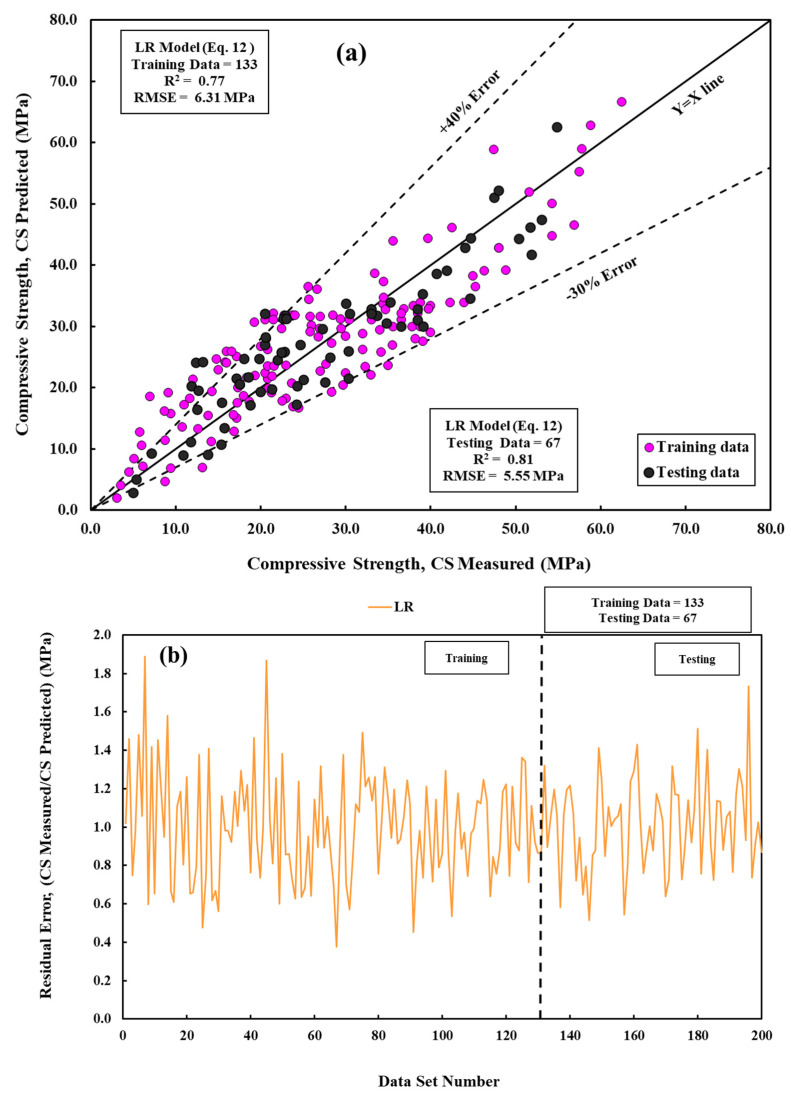
A comparative of measured and predicted CS is conducted using the LR model across both training and testing datasets. (**a**) Modeling and (**b**) residual error assessment.

Based on the result obtained, the cement mortar compressive strength can be predicted with R^2^ of 0.77, as shown in Figure 4a. Based on the developed model parameters, the cement/sand ratio, followed by w/c and Al_2_O_3_, has the highest impact on the compressive strength of cement. The residual error is shown in Figure 4b.

### 3.2. Pure Quadratic Model

**Figure 5:**
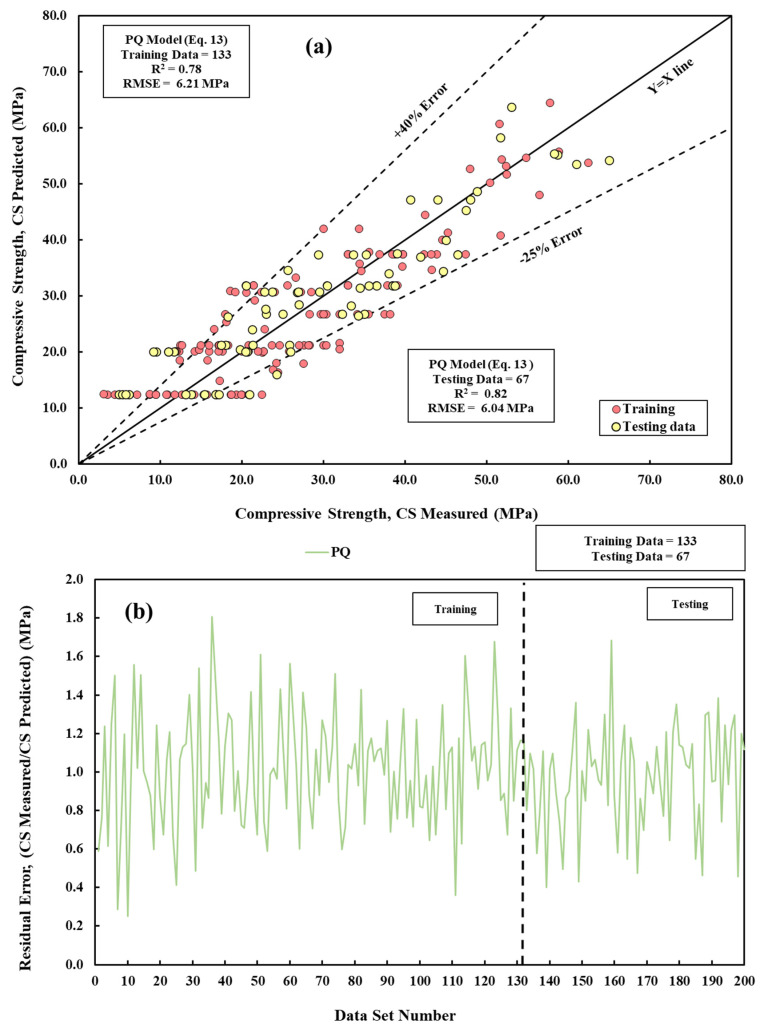
A comparison of measured and predicted CS is conducted using the PQ model across both training and testing datasets. (**a**) Modeling and (**b**) residual error assessment.

A pure quadratic or quadratic regression model describes the connection between the CS of cement mortar and w/c, t, c/s, curing temperature, water pH, and chemical composition of cement using a quadratic equation (Equation (13)), as shown in Figure 5a,b.

(13) $$\begin{array}{cc} CS\;=584+0.003\left(\frac{c}{s}\right) & +\;0.3\left(t\right)-0.002\;\left(\frac{c}{s}\right)-56\;\left(T\right)+2.60\;\left(pH\right)-11.5\;\left({SiO}_{2}\right)+7\left(CaO\right)-6.8\;\left({Al}_{2}O_{3}\right)-4.7\;\left({Fe}_{2}O_{3}\right)\\ & -0.006\;{\left(t\right)}^{2}+1.34\;{\left(T\right)}^{2}{-\;0.0015\;\left(pH\right)}^{2}{+\;0.25\;\left({SiO}_{2}\right)}^{2}-0.063\left(CaO\right)-0.007{{(Fe}_{2}O_{3})}^{2} \end{array}$$

### 3.3. Interaction Model

In statistical analysis and regression modeling, an interaction model is a model that incorporates interaction terms involving two or more independent variables such as CS, t, c/s, curing temperature, pH of water, and the chemical composition of cement. Interaction terms contain the collective impact or influence of many variables on the CS of cement mortar, and they determine if the correlation between one independent variable (input variables) and the dependent variable (compressive strength) is contingent upon the magnitude of another independent variable (Equation (14)).

(14) $$\begin{array}{cc} CS\;=\;419.4 & +\;75.8\;\left(w/c\right)-4.43\;\left(t\right)-23.16\;\left(c/s\right)-22.2\;\left(T\right)-0.175\;\left(pH\right)+0.47\;\left({SiO}_{2}\right)+\;3.8\left(CaO\right)\\ & -104\left({Al}_{2}O_{3}\right)+61.8\left({Fe}_{2}O_{3}\right)+1.23\left(w/s\right)\left(t\right)-3.22\left(w/s\right)\left({SiO}_{2}\right)-2.07\beta_{16}\left(w/s\right)\left(CaO\right)\\ & +11.4\;\left(w/s\right)\left({Fe}_{2}O_{3}\right)+0.32\;\left(t\right)\left(c/s\right)-0.024\left(t\right)\left(T\right)+0.026\left(t\right)\left(pH\right)+0.020\left(t\right)\left({SiO}_{2}\right)\\ & +0.055\left(t\right)\left(CaO\right)+0.032\left(t\right)\left({Al}_{2}O_{3}\right)+0.013\left(t\right)\left({Fe}_{2}O_{3}\right)+0.0009\left(c/s\right)\left(T\right)+0.07\left(c/s\right)\left(pH\right)\\ & +2.42\left(c/s\right)\left({SiO}_{2}\right)+0.19\left(c/s\right)\left(CaO\right)-0.083\left(c/s\right)\left({Al}_{2}O_{3}\right)+0.00007\left(c/s\right)\left({Fe}_{2}O_{3}\right)\\ & +0.05\left(T\right)\left(pH\right)+0.087\left(T\right)\left({SiO}_{2}\right)-0.003\left(T\right)\left(CaO\right)+2.64\left(T\right)\left({Al}_{2}O_{3}\right)+2.11\left(T\right)\left({Fe}_{2}O_{3}\right)\\ & +0.001\left(pH\right)\left({SiO}_{2}\right)+0.001\left(pH\right)\left(CaO\right)-0.09\left({SiO}_{2}\right)\left(CaO\right)+0.005\left({SiO}_{2}\right)\left({Al}_{2}O_{3}\right)\\ & -0.043\;\left({SiO}_{2}\right)\left({Fe}_{2}O_{3}\right)+0.65\left(CaO\right)\left({Al}_{2}O_{3}\right)-1.76\left(CaO\right)\left({Fe}_{2}O_{3}\right)+0.4\left({Al}_{2}O_{3}\right)\left({Fe}_{2}O_{3}\right) \end{array}$$

**Figure 6:**
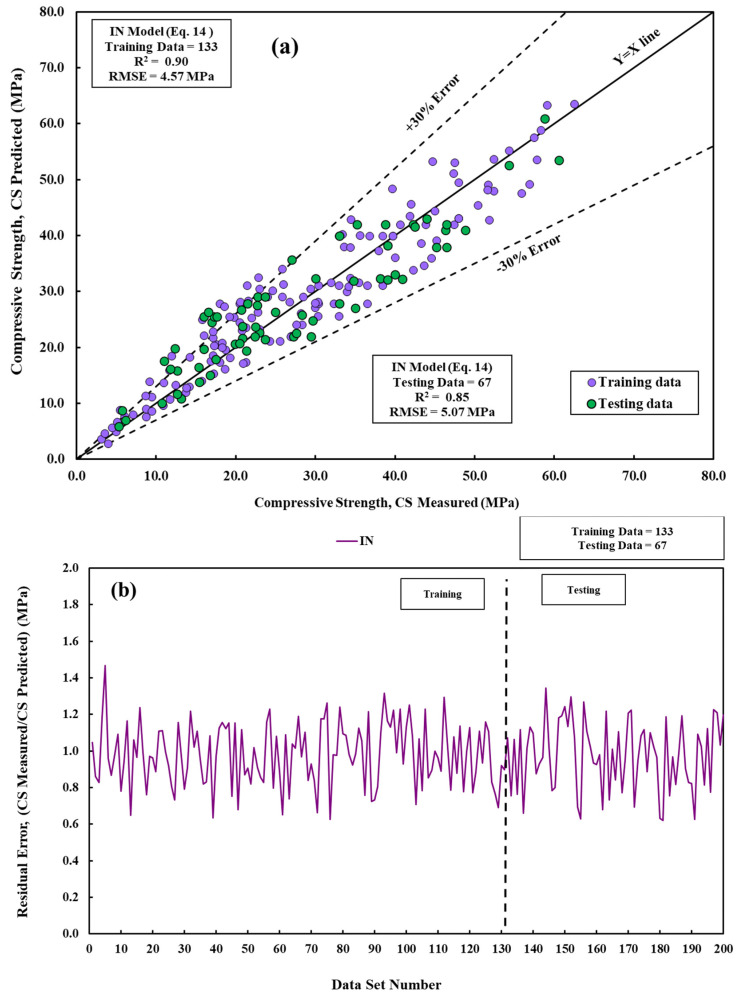
A measured and predicted CS is compared using the IN model across training and testing datasets. (**a**) Modeling and (**b**) residual error assessment.

Compared to linear and pure quadratic models, the interaction model predicts the compressive strength of cement mortar very well (Figure 6).

### 3.4. M5P Tree Model

**Figure 7:**
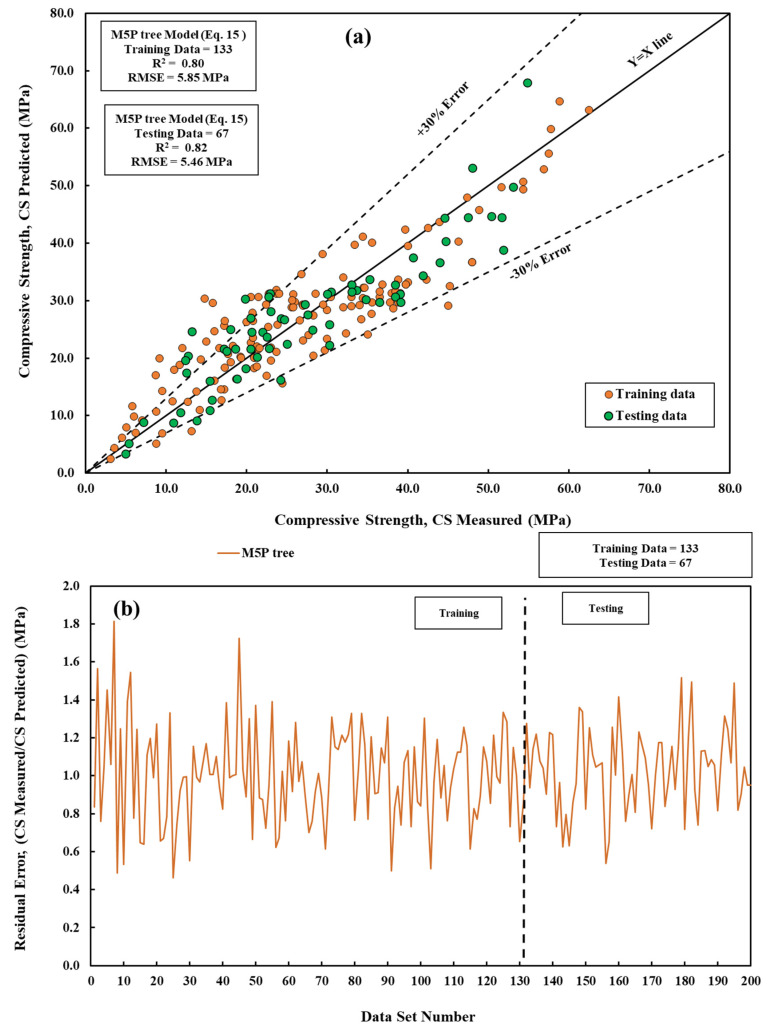
A comparative of measured and predicted CS is conducted using the M5P tree model across both training and testing datasets. (**a**) Modeling and (**b**) residual error assessment.

In this study, a novel M5P tree model was developed with an established value of m. This model was utilized to forecast the compressive strength of cement mortar using a dataset of 200 mix-design data points. To assure the stability and reliability of the model, two-thirds of the dataset was randomly chosen for training, while the remaining one-third was set aside for evaluating the model’s ability to make accurate predictions. The training and testing sets underwent a thorough evaluation utilizing multiple metrics to assess their prediction accuracy, such as R^2^, MAE, and RMSE. The equation *Y* = *b*_0_ + *b*_1_ × *X*_1_ + *b*_2_ × *X*_2_ represents these functions, where *b*_0_, *b*_1_, and *b*_2_ are constants in linear regression that indicate the model parameters. Figure 7 depicts the correlation between the anticipated and actual compressive strength. Significantly, the dataset displays a line representing an error margin of 30%, which means that all recorded values are within this range. The model coefficient of determination, R^2^, is calculated to be 0.82, indicating that it performs better than the LR model [57,58,59].

(15) $$CS=-18.3\;\times \;\frac{w}{c}+0.1\;\times \;t+\;43.5\;\times \;\frac{c}{s}\;-\;3.35\times \;{SiO}_{2}-\;1.4\;\times \;CaO-\;3.9\;{\times \;Fe}_{2}O_{3}+197.2$$

### 3.5. Artificial Neural Network (ANN)

**Figure 8:**
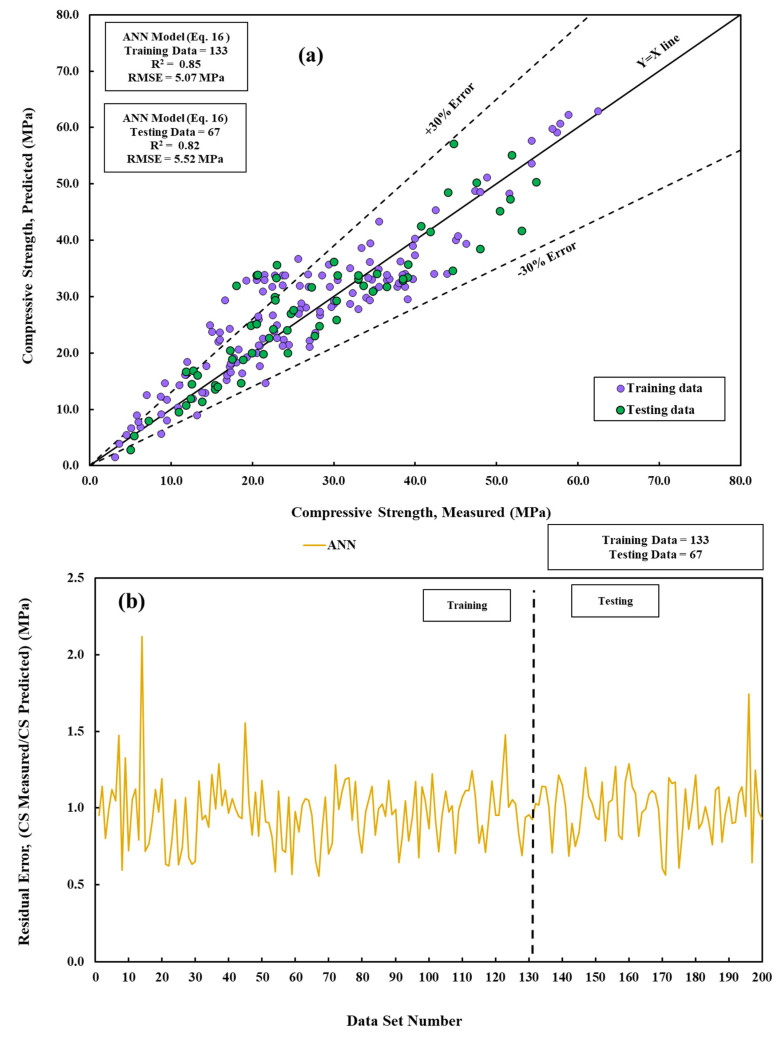
A comparative of measured and predicted CS is conducted using the ANN model across training and testing datasets. (**a**) Modeling and (**b**) residual error assessment.

Developing an artificial neural network (ANN) model is a step-by-step process that requires setting the number of neurons in the hidden layer, the learning rate, momentum, and the number of iterations. This study utilized seven neural networks to represent the hidden layers. The learning rate, training length, and momentum were assigned the values of 0.1, 5000, and 0.1, respectively. The number of epochs, a hyperparameter, determines the maximum number of iterations the learning algorithm goes through on the training dataset. As the number of epochs increases, the R^2^ values also grow while the RMSE values fall. This suggests a reduction in errors. Figure 8 depicts a comparison of projected and absolute CS values. The selected input variables should encompass all essential information related to the target values. This study examined nine specific parameters for assessing the compressive strength of cement mortar [58].

(16) $$\begin{array}{cc} CS=\frac{-3.67}{1+e^{-\alpha 1}} & +\frac{-3.18}{1+e^{-\alpha 2}}+\frac{2.05}{1+e^{-\alpha 3}}+\frac{2.10}{1+e^{-\alpha 4}}\frac{2.02}{1+e^{-\alpha 5}}+\frac{-1.2}{1+e^{-\alpha 6}}\frac{-2.56}{1+e^{-\alpha 7}}+\frac{-2.98}{1+e^{-\beta 8}}\frac{-1.76}{1+e^{-\alpha 9}}\\ & +\frac{-1.77}{1+e^{-\alpha 10}}\frac{2.68}{1+e^{-\alpha 11}}+\frac{2.69}{1+e^{-\alpha 12}}\frac{2.30}{1+e^{-\alpha 13}}+\frac{-2.42}{1+e^{-\alpha 14}}+\frac{-2.59}{1+e^{-\alpha 15}}\frac{1.94}{1+e^{-\alpha 16}}+0.34 \end{array}$$

$$\left[\begin{array}{cccccccccccc} -0.28 & -1.67 & -0.35 & 2.91 & 2.15 & 2.38 & 1.92 & 0.63 & 0.28 & -2.43 & 2.66 & -2.12\\ 3.99 & 1.15 & 3.68 & 1.53 & 2.19 & 1.51 & -6.09 & 2.75 & 6.63 & 6.54 & 0.10 & 1.0\\ 3.16 & -0.30 & 2.65 & -0.33 & -3.31 & 11.3 & 4.87 & 0.66 & 4.17 & -4.41 & -2.18 & 2.14\\ 0.97 & 0.23 & 0.49 & 2.19 & 8.08 & 0.58 & -2.24 & 4.02 & 0.08 & -0.76 & 0.02 & -1.27\\ 2.25 & -1.92 & -1.80 & 4.78 & -0.87 & 6.34 & 4.21 & -2.79 & 8.91 & -3.63 & 1.02 & -1.07\\ 0.10 & 0.64 & 0.38 & 0.96 & 0.54 & 1.94 & 1.10 & -0.26 & 0.34 & 0.001 & -1.52 & -1.88\\ 1.99 & 1.77 & 4.59 & 6.76 & 3.54 & 2.98 & -7.16 & 1.06 & 0.48 & -1.56 & -0.09 & -3.96\\ 1.61 & 0.17 & 1.10 & 2.24 & 0.89 & 2.56 & 0.51 & 0.83 & -0.69 & 0.49 & 0.003 & -1.69\\ -0.62 & 3.94 & -0.39 & 0.95 & 0.045 & -1.70 & 5.88 & -4.03 & -0.15 & -3.77 & -0.75 & -3.26\\ 0.29 & 3.16 & 1.73 & -0.12 & -0.067 & -0.68 & 1.91 & -0.086 & -1.19 & 2.61 & -0.56 & -2.55\\ -1.41 & 1.44 & 1.34 & 3.32 & 1.74 & -2.12 & -0.40 & -0.37 & -1.19 & 6.25 & -5.48 & -2.97\\ -0.71 & 1.43 & -2.24 & 3.64 & 6.80 & 6.33 & -0.77 & 5.49 & 0.24 & 6.13 & 0.92 & 0.49\\ 2.50 & 0.50 & 3.81 & -0.37 & -2.22 & 2.70 & -0.21 & -0.23 & 3.07 & 1.16 & -0.74 & -1.43\\ 2.10 & -2.01 & -2.29 & -4.87 & -2.55 & 3.26 & -0.67 & -0.25 & 3.53 & -10.3 & -3.07 & -12.1\\ 1.55 & -2.27 & -2.66 & -7.63 & 2.39 & 2.89 & -1.32 & 1.68 & 2.30 & 1.83 & 0.44 & -2.81\\ -0.02 & -0.008 & -0.041 & -2.28 & -0.58 & 0.22 & 3.26 & 1.58 & -0.95 & -1.73 & -0.44 & -2.16 \end{array}\right]\;\times \;\left[\begin{array}{c} w/c\\ t\\ c/s\\ T\\ pH\\ {SiO}_{2}\\ CaO\\ {Fe}_{2}O_{3}\\ {Al}_{2}O_{3}\\ 1\\ 1\\ 1 \end{array}\right]\;=\;\left[\begin{array}{c} \alpha 1\\ \alpha 2\\ \alpha 3\\ \alpha 4\\ \alpha 5\\ \alpha 6\\ \alpha 7\\ \alpha 8\\ \alpha 9\\ \alpha 10\\ \alpha 11\\ \alpha 12\\ \alpha 13\\ \alpha 14\\ \alpha 14\\ \alpha 16 \end{array}\right]$$

## 4. Statistical Assessment

**Figure 9:**
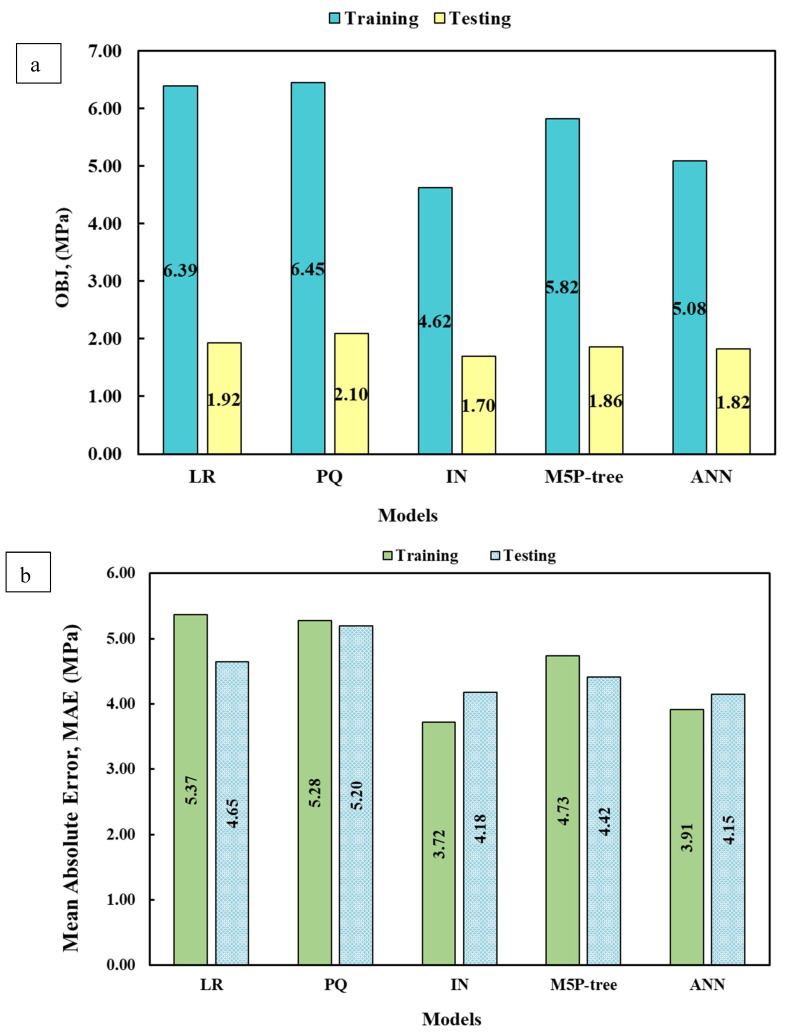

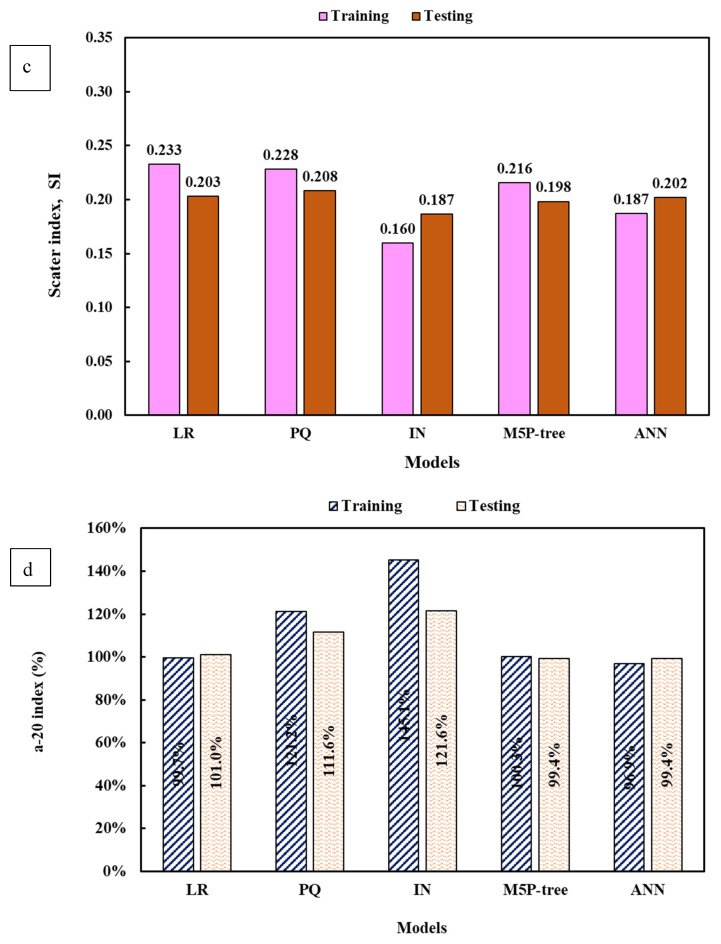
The statistical assessment of the developed models. (**a**) OBJ, (**b**) MAE, (**c**) SI, and (**d**) a-20 index.

Different statistical assessments were used to evaluate the developed models’ predictions and compare the developed models’ results. The residual error of the developed models shows that the error range in the prediction was less than 2.5 MPa, as shown in Figure 8. The OBJ values of the training and testing data set are shown in Figure 9a. The OBJ value of the linear model was 6.39 MPa and 1.92 MPa for the training and testing data, respectively. With 6.45 MPa, the OBJ of the pure quadratic model was the highest. The interaction model has the lowest OBJ value at 27% and 28.3% less than the linear and pure quadratic models.

Figure 9b shows the developed models’ mean absolute error (MAE). The interaction model has the lowest MAE with 3.72 MPa and 4.18 for the training and testing data set. The MAE for the linear and pure quadratic models were near each other, with 5.37 MPa and 5.28 MPa (Figure 9b).

The normalized error measurement is known as the scatter index (SI), and is given as a percentage. The value of the scatter index of the developed model is shown in Figure 9c. Based on the SI values, the interaction model is more reliable in predicting cement mortar CS than linear and pure quadratic models.

**Figure 10:**
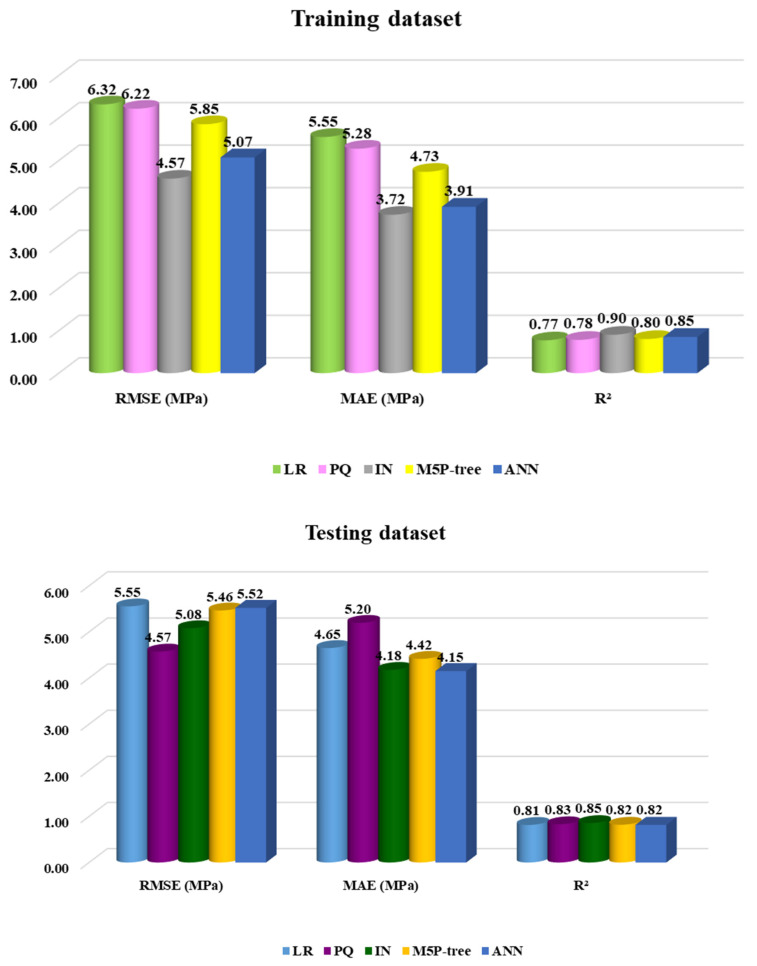
The accuracy of the proposed models.

The number of samples that fit the prediction values with a variance of ±20% from the experimental values is displayed by the a-20 index-evaluated measure. To put it another way, the a-20 metric calculates the percentage of instances in which the absolute difference between the actual and projected values is 20% or less of the actual value. Since the model may produce more precise predictions closer to the actual value, a higher a-20 score denotes better predictive accuracy. Also, based on a-20 index values, the interaction model will be used to predict the cement mortar CS, as shown in Figure 9d. The R^2^ and RMSE of the developed models are shown in Figure 10.

## 5. Correlation between Compressive with Flexural and Tensile Strengths

**Figure 11:**
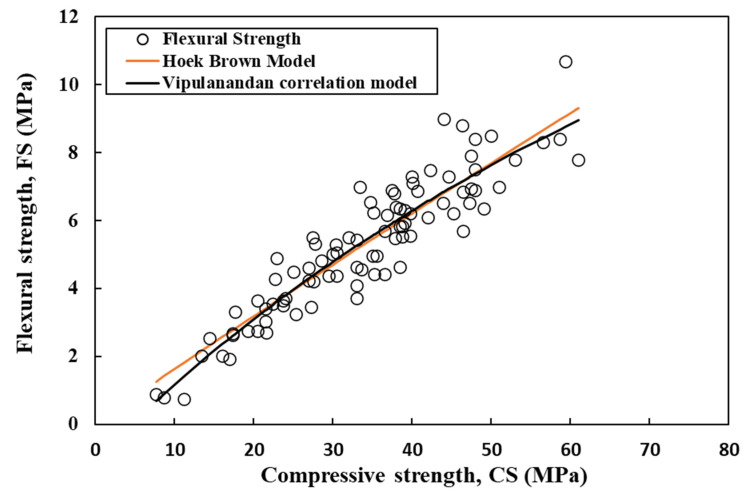
Correlation between compressive strength and the flexural strength of cement mortar using Vipulanandan and Hoek–Brown models.

**Figure 12:**
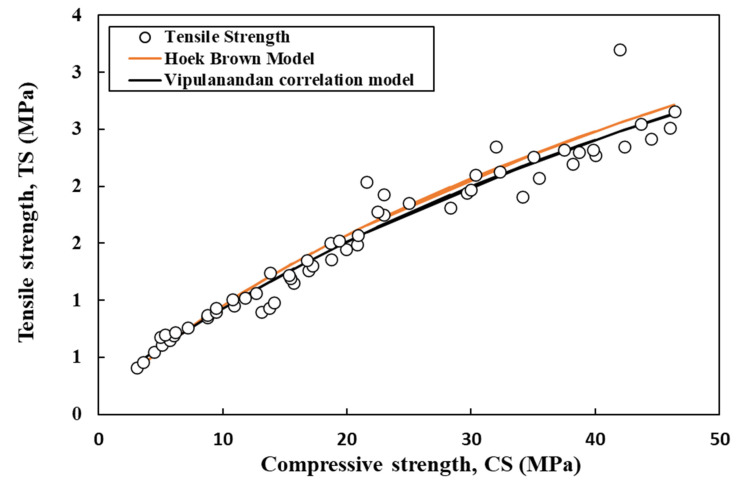
Correlation between compressive strength and the tensile strength of cement mortar using Vipulanandan and Hoek–Brown models.

The flexural and tensile strength of cement mortar were estimated using the Vipulanandan correlation model and Hoek–Brown model (Equations (17) and (18)) [60,61,62], which relates the estimated strengths to the compressive strength. This relationship is illustrated in Figure 11 and Figure 12. With a rise in compressive strength from 7 MPa to 45 MPa, the flexural and tensile strength of the cement mortar also increased from 1 MPa to 7.6 MPa and from 0.5 MPa to 2.3 MPa, respectively (Figure 11 and Figure 12).

(17a) $$FS=-1.45+\;\frac{CS}{4.21+0.021\;CS}\;$$

(17b) $$FS=CS-(0.003\times (\frac{-0.79\;CS}{-0.003}+2.05))$$

where *FS* is the flexural strength and *CS* is the compressive strength.

(18a) $$TS=0.2+\;\frac{CS}{12.45+0.14\;CS}$$

(18b) $$TS=CS-(0.0009\times \left(\frac{-0.72\;CS}{-0.0009}-18.7\right))$$

where *TS* is the tensile strength and *CS* is the compressive strength.

## 6. Sensitivity Analysis

**Figure 13:**
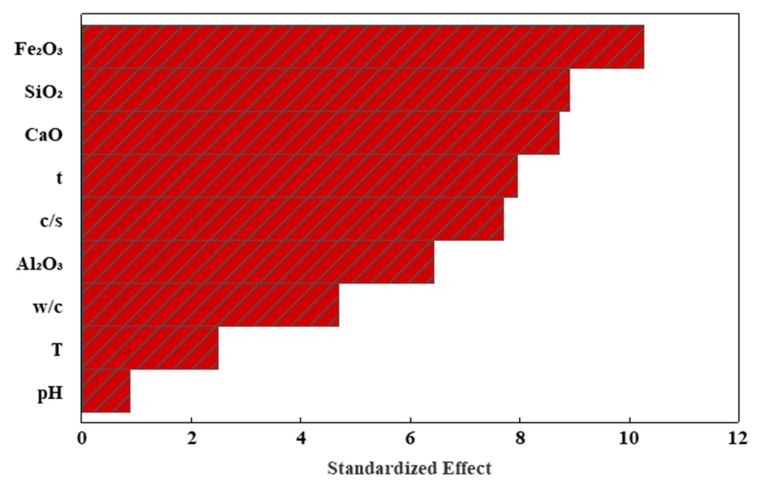
Sensitivity analysis using Pareto chart.

A sensitivity analysis was conducted using a Pareto chart to evaluate each independent variable’s influence on cement mortar’s compressive strength value (Table 3). A Pareto chart is an effective instrument that facilitates comprehension of the primary elements that have the most significant impact on the dependent variable, which is the compressive strength of cement mortar. Based on the Pareto chart analysis, iron oxide has a high effect on the compressive strength of cement mortar, followed by silicon dioxide and calcium dioxide, respectively, as shown in Figure 13.

## 7. Limitations of the Study

1. The study relies on data from 200 cement mortar experiments, which, although substantial, may need to be more exhaustive to encompass all the differences and intricacies in cement mortar behavior. An expanded dataset has the potential to yield more reliable and widely applicable findings;
2. The findings are derived from precise test settings and mixtures. Due to variations in local materials, ambient conditions, and construction procedures, the results may not apply to other areas or situations;
3. Various modeling approaches were used, including linear regression, pure quadratic interaction, M5P tree, and artificial neural networks. However, it is important to note that each methodology has specific assumptions and limits. It is not possible for any one model to completely understand and explain the intricate behavior of cement mortar in tile adhesive applications in all situations;
4. The study primarily focuses on the exact chemical compositions of silicon dioxide, calcium dioxide, iron (III) oxide, aluminum oxide, and the pH value. Other potentially relevant components or additions in cement mortar were unaccounted for, which might impact the overall knowledge of material behavior;
5. The long-term environmental consequences, such as freeze-thaw cycles, chemical exposure, and other durability factors, that might potentially affect the performance of cement mortar over lengthy periods;
6. Empirical equations are mathematical equations derived from observation or experimentation rather than theoretical principles;
7. The curing conditions employed in the experiments, such as temperature and humidity, could not encompass the whole spectrum of real-world circumstances, impacting the prediction models’ precision and relevance;
8. The data obtained from prior research may exhibit discrepancies or changes in measuring methodologies, which might impact the reliability and precision of the analysis and subsequent models;
9. Validation measures, such as determination coefficient, mean absolute error, root-mean-square error, objective function, scatter index, and a-20 index, were used. However, further validation using external datasets might enhance the dependability of the models.

## 8. Conclusions

The following conclusions are drawn based on the data gathered from various research investigations and the simulation of the compressive strength of cement mortar at 200 different water/cement (w/b), cement/sand (c/s), curing temperature (T), water pH, the different chemical composition of cement, and curing time.

1. Silicon and calcium dioxide were tested up to 30% and 76.5%, respectively. The pH of water curing varied between 3.5 and 7.6. The medium values of the iron and aluminum oxides were 4.51% and 5.55%, respectively;
2. According to the dataset gathered, the water/cement ratio, cement/sand ratio, and curing duration varied from 0.3 to 1.2, 0.14 to 0.408, and 1 day to 365 days. An ideal ratio of 0.34 cement to sand is recommended for achieving optimal compressive strength in cement mortar;
3. The interaction model was employed to forecast the strength of cement mortar. It was trained using 2/3 of the 200 data points collected from the literature. The IN model accurately predicted the CS of the testing data, achieving a high coefficient of determination (R^2^ = 0.90). Both the linear relation (LR) and pure quadratic (PQ) models were developed using identical variables;
4. In addition to the conventional curing period, this research’s findings demonstrate that the IQ model can forecast the cement mortar’s CS. The PQ models accurately predicted the CS based on the training and testing datasets, outperforming other models regarding R^2^, RSME, MAE, OBJ, SI, and the a-20 index;
5. Based on the Vipulanandan correlation model and the Hoek–Brown model, the flexural and tensile strengths of the cement mortar can be predicted as a function of the compressive strength;
6. According to the Pareto chart, the cement chemical compositions have a high effect on the compressive strength of cement mortar.

## Figures and Tables

**Table 1:** Summary of the collected dataset used for modeling.

Variables	References										
	[7]	[23]	[24]	[25]	[26]	[27]	[28]	[29]	[30]	[31]	
Water-to-cement ratio, w/c	0.5	0.4	0.3–0.65	0.5	0.5	0.56	0.5–1.2	1	0.4	0.5	Ranged between 0.3–1.2
Curing time, t (days)	7–365	1–90	3, 7, 28	2, 28	7, 28	7, 28, 90	28	7, 14, 28	7, 28	7, 28	Ranged between 1–365
Cement-to-sand ratio, c/s	0.333	0.333	0.33–0.5	0.333	0.408	0.333	0.14–0.33	0.333	-	0.333	Ranged between 0.14–0.408
Temperature (T °C)	20, 23	20	20	20	20	22	20	20	--	20	Ranged between 20–23
pH value	3.5, 7	7	7.85	7	7	7	7	7	-	7	Ranged between 3.5–7.85
Silicon dioxide, SiO_2_ (wt %)	13.48	20.33	18.91	18.95	22	20.8	30	18.91	20.5	-	Varied between 13.48–30
Calcium dioxide, CaO (wt %)	67.46	69.93	66.67	63.83	60.1	61.94	42	63.83	62.3	-	Ranged between 42–69.93
Iron oxide, Fe_2_O_3_ (wt %)	7.28	5.56	6.5	4.5	2.1	4	5.55	4.94	3.4	-	Varied between 2.1–7.28
Aluminum oxide, Al_2_O_3_ (wt %)	3.69	4.2	4.51	5.89	6.6	5.52	8.5	4.51	4	-	Ranged between 3.69–8.5
Compressive strength, CS (MPa)	34.47–54.85	13.21–24.45	8.5–46.4	39.1–53	18.3–25.6	27–40.7	3.1–23	4–7	35.15–37.72	30.7–41.6	Ranged between 3.1–54.85
Flexural strength, FS (MPa)	-	-	-	6.3–7.8	-	4.6–6.87	-	-	6.23–6.81	3.92	Ranged between 3.92–7.8
Tensile strength, TS (MPa)	-	-	1.85–2.51	-	-	-	0.41–1.78	-	-	-	Ranged between 0.41–2.51

**Table 2:** An overview of the statistical analysis conducted on the mortar mixtures.

Variables	w/c	T (days)	c/s	SiO_2_ (%)	CaO (%)	Fe_2_O_3_ (%)	Al_2_O_3_ (%)	CS (MPa)
Mean	0.55	28.80	0.35	20.20	63.26	5.22	5.26	27.19-
Median	0.50	28.00	0.33	18.91	66.67	4.51	5.55	24.84
Mode	0.50	28.00	0.33	18.91	66.67	4.51	6.50	33.01
SD	0.20	48.05	0.09	5.28	11.01	1.85	1.28	13.13
Var	0.04	2308.55	0.01	27.90	121.31	3.44	1.63	172.43
Kurt	2.81	24.38	1.98	−0.23	−0.04	−0.70	0.28	−0.38
Skew	1.82	4.44	0.34	0.72	−0.96	0.69	−0.44	0.44
Min	0.30	1.00	0.14	13.48	42.00	3.00	1.20	3.11
Max	1.20	365.00	0.408	30.00	76.50	8.71	7.78	62.50
No. of data	278	280	275	261	242	266	266	280

**Table 3:** A summary of the criteria used to evaluate the efficiency of the developed models.

Model	Fig. (No)	Eq. (No.)	Training			Testing			Ranking
			RMSE (MPa)	MAE (MPa)	R^2^	RMSE (MPa)	MAE (MPa)	R^2^	
LR	4	12	6.32	5.55	0.77	5.55	4.65	0.81	4
PQ	5	13	6.22	5.28	0.78	4.57	5.20	0.83	5
IN	6	14	4.57	3.72	0.90	5.08	4.18	0.85	1
M5P-tree	7	15	5.85	4.73	0.80	5.46	4.42	0.82	3
ANN	8	16	5.07	3.91	0.85	5.52	4.15	0.82	2

## Data Availability

The authors will make the raw data supporting this article’s conclusions available upon request.
